# Increased risk of major adverse cardiovascular events in patients with deep and infected diabetes-related foot ulcers

**DOI:** 10.1007/s00125-024-06316-z

**Published:** 2024-11-07

**Authors:** Nick S. R. Lan, Jonathan Hiew, Ivana Ferreira, J. Carsten Ritter, Laurens Manning, P. Gerry Fegan, Girish Dwivedi, Emma J. Hamilton

**Affiliations:** 1 Centre of Excellence for Cardiometabolic Health, Fiona Stanley Hospital, Perth, Australia; 2 Department of Cardiology, Fiona Stanley Hospital, Perth, Australia; 3 Medical School, The University of Western Australia, Perth, Australia; 4 Harry Perkins Institute of Medical Research, Perth, Australia; 5 Centre of Excellence Multidisciplinary Diabetes Foot Ulcer Service, Fiona Stanley and Fremantle Hospitals Group, Perth, Australia; 6 Department of Podiatry, Fiona Stanley Hospital, Perth, Australia; 7 Medical School, Curtin University, Perth, Australia; 8 Department of Vascular Surgery, Fiona Stanley Hospital, Perth, Australia; 9 Department of Infectious Diseases, Fiona Stanley Hospital, Perth, Australia; 10 Department of Endocrinology and Diabetes, Fiona Stanley Hospital, Perth, Australia

**Keywords:** Cardiovascular diseases, Coronary artery disease, Diabetes, Foot ulcer, Myocardial infarction, Risk factors

## Abstract

**Aims/hypothesis:**

Diabetes-related foot ulceration (DFU) is associated with increased cardiovascular risk, but the mechanisms remain unclear. Inflammation and infection are mediators of CVD, which may be important in DFU.

**Methods:**

Prospectively collected data from patients attending a multidisciplinary DFU service were analysed. A deep ulcer was defined as one that reached muscle, tendon or deeper structures. Patients were categorised into four DFU groups: not deep and no infection (D−/I−), not deep but infected (D−/I+), deep with no infection (D+/I−) or deep with infection (D+/I+). Incident major adverse cardiovascular events (MACE) were defined as hospitalisation for myocardial infarction, stroke or transient ischaemic attack, or heart failure. Survival analyses were performed using the logrank test and multivariate Cox regression.

**Results:**

Of 513 patients, 241 (47.0%) were in the D−/I− group, 110 (21.4%) were in the D−/I+ group, 35 (6.8%) were in the D+/I− group and 127 (24.8%) were in the D+/I+ group. MACE or all-cause mortality occurred in 75 patients (14.6%), and MACE alone occurred in 46 patients (9.0%) after median follow-up of 381 days (IQR 220–551) and 404 days (IQR 228–576), respectively. Infection was associated with significantly higher MACE or all-cause mortality (21.5% vs 8.7%; *p*<0.001) and MACE alone (13.5% vs 5.1%; *p*=0.003). MACE or all-cause mortality was significantly higher in the D+/I+ group (D−/I− 7.9%; D−/I+ 15.5%; D+/I− 14.3%; D+/I+ 26.8%; *p*<0.001), as was MACE alone (D−/I− 5.0%; D−/I+ 10.9%; D+/I− 5.7%; D+/I+ 15.7%; *p*=0.017). Infection and a deep ulcer were independent predictors of adverse outcomes.

**Conclusions/interpretation:**

Deep and/or infected DFUs are associated with increased cardiovascular risk compared with DFUs that are not deep or infected. These findings provide a potential mechanistic explanation that requires investigation.

**Graphical Abstract:**

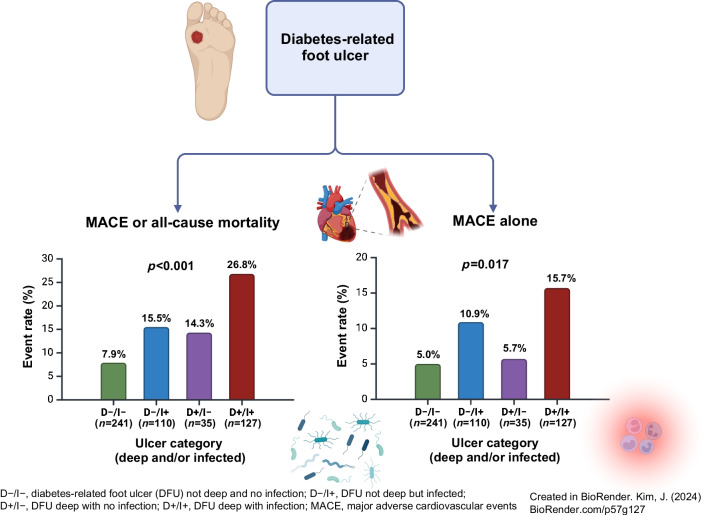

**Supplementary Information:**

The online version contains supplementary material available at 10.1007/s00125-024-06316-z.


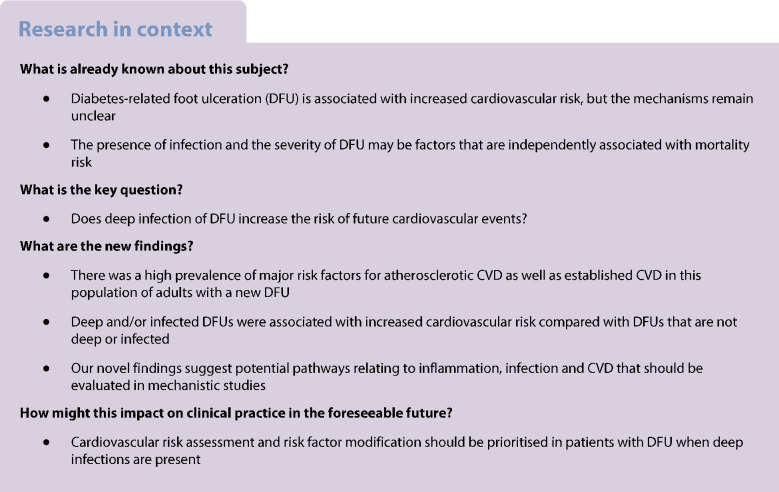


## Introduction

CVD is a leading cause of morbidity and mortality among people with a history of diabetes-related foot ulceration (DFU) [1–3]. Whilst diabetes mellitus is associated with elevated cardiovascular risk, studies have indicated that people with DFU face an even greater susceptibility to major adverse cardiovascular events (MACE) [4]. Crucially, the underlying mechanisms driving the increased cardiovascular risk in people with DFU remain poorly understood. People with DFU often have a greater burden of cardiovascular risk factors and diseases, such as peripheral arterial disease (PAD), that contribute to a heightened risk of MACE [5, 6]. Furthermore, peripheral neuropathy is a microvascular complication of diabetes that plays a critical role in the pathogenesis of DFU, and is associated with increased cardiovascular risk [7, 8]. Nonetheless, it is plausible that factors specific to the DFU may also increase cardiovascular risk [9]. The presence of infection and severity of DFU are factors that are independently associated with mortality, but studies linking ulcer characteristics with incident MACE are lacking [10–14].

Inflammation and immunity are critical mediators of atherosclerotic CVD and heart failure (HF) [15, 16]. Inflammatory conditions such as rheumatoid arthritis, psoriatic arthritis, gout and inflammatory bowel disease are associated with an increased risk of MACE [17–19]. In addition, infections such as pneumonia, COVID-19 and HIV increases cardiovascular risk [20–22]. Whilst diabetes is itself an inflammatory condition, there is evidence that DFU further increases inflammation; studies have shown greater expression of acute-phase proteins, cytokines and chemokines in DFU, correlating with ulcer severity [23–26]. Moreover, many DFUs become infected, manifesting as local infection involving the skin and subcutaneous tissue, deeper infection affecting muscle, tendon or bone (i.e. osteomyelitis) and/or systemic infection [27]. Superimposed infection of DFU may result in heightened systemic inflammation and immune system activation, which may lead to atherosclerotic CVD and HF [9, 28].

However, to our knowledge, the link between inflammation/infection and incident MACE in people with DFU has never been studied. We hypothesised that deep infection of DFU is associated with incident MACE due to heightened systemic inflammation, providing a mechanistic explanation for the increased cardiovascular risk observed in people with DFU.

## Methods

Adult patients (aged over 18 years) with a new DFU who received treatment at a multidisciplinary DFU service within a Western Australia tertiary hospital group between January 2022 and April 2024 were identified. This hospital group is one of three tertiary hospital groups for Western Australia, and, at the time of the study, received all referrals for patients with DFU within its catchment area. The DFU service is staffed by a team of endocrinologists, infectious diseases specialists, vascular surgeons, podiatrists and an orthotist, and is a National Association of Diabetes Centres Centre of Excellence High-Risk Foot Service. Standardised data for all new patients with DFU and any existing patients with a new DFU who attended the service since January 2022 have been prospectively captured as part of the Australian Diabetes Foot Registry. The registry enables audits and benchmarking to be undertaken by the National Association of Diabetes Centres and the Australian Diabetes Society. Data for the electronic registry are collected and entered into the database by clinicians within the DFU service to ensure accuracy. Data pertaining to patient demographics, diagnosed comorbidities, medications prescribed, pathology results and wound characteristics were extracted from the local database and supplemented by a review of electronic medical records and hospital discharge summaries. Patient sex and ethnicity were self-reported at the time of hospital presentation. Patients incorrectly classified as having DFU or with incomplete data regarding wound depth and infection were excluded from the analysis. Social deprivation was determined using the Australian Socio-Economic Indexes for Areas, whereby socioeconomic advantage and disadvantage are categorised into quintiles according to the patient’s residential postcode [29]. This study was approved by the local human research ethics committee and local institution (SMHS RGS7053/RGS4326 and FSFHG53018) and was performed in accordance with the principles of the Declaration of Helsinki.

At the multidisciplinary DFU service, the standard of care is to comprehensively assess both patient and DFU characteristics, including neurovascular assessment (peripheral pulses, toe pressures, sensation using monofilaments and/or Ipswich touch test) and wound measurement (using a Silhouette Star camera; ARANZ Medical, New Zealand) with depth assessment/probe-to-bone test and classification using wound classification systems [30]. For this study, the infection and depth variables from the SINBAD classification system (site, ischaemia, neuropathy, bacterial infection, area and depth: see ESM Table 1) were used for analyses [30]. For patients with multiple DFUs, only data pertaining to the most clinically important ulcer were used. The cohort was divided into four groups according to DFU depth and infection severity as per the SINBAD score categories: (1) not deep and no infection (D−/I−); (2) not deep but infected (D−/I+); (3) deep with no infection (D+/I−); and (4) deep with infection (D+/I+). A deep ulcer was defined as reaching muscle, tendon or deeper structures. Foot infection was defined as signs or symptoms of bacterial infection, either local (skin, subcutaneous tissue or deeper) or systemic. Local infection required as least two of the following characteristics: (1) local swelling or induration; (2) erythema at least 0.5 cm around the ulcer; (3) local tenderness or pain; (4) local warmth; and (5) purulent discharge [30].

The co-primary outcomes of interest were: (1) a composite of first occurrence of incident MACE or all-cause mortality and (2) incident MACE alone. MACE was defined as the first occurrence of hospitalisation for myocardial infarction, stroke or transient ischaemic attack, or HF, including fatal and non-fatal occurrences, and was determined using hospital discharge summaries and medical records. Ulcer status at the time of the event or the end of follow-up was also determined.

Statistical analyses were performed using SAS version 9.4 (SAS Institute, USA) and SPSS version 29 (IBM, USA). Descriptive data are presented using mean ± SD, count (%) or median (IQR) as appropriate. Differences between groups were compared using an unpaired Student’s *t* test, the Wilcoxon rank-sum test, Pearson’s *χ*^2^ test or Fisher’s exact test as appropriate. Kaplan–Meier survival curves were generated, and time-to-event data were compared using the logrank test. Cox proportional hazards regression analysis was performed to estimate HRs and 95% CI for the outcomes of interest. Multivariate Cox regression analyses were performed using models that were: (1) unadjusted; (2) adjusted for age, social deprivation score, type 1 diabetes, hypertension, dyslipidaemia, ever-smoking history, use of sodium-glucose cotransporter 2 (SGLT2) inhibitors or glucagon-like peptide-1 (GLP1) agonists; (3) adjusted for covariates in model 2 plus diagnosed atherosclerotic CVD (either CHD, PAD or prior stroke or transient ischaemic attack), HF, nephropathy and number of other comorbidities (chronic obstructive pulmonary disease, depression and osteoporosis); and (4) adjusted for covariates in model 2 and 3 plus wound area (≥1 cm^2^ vs <1 cm^2^) and location (midfoot/hindfoot vs forefoot). A two-tailed *p* value <0.05 was defined as statistically significant.

## Results

Of 557 patients identified, 36 (6.5%) with Charcot foot but no DFU and eight (1.4%) with a SINBAD wound classification score of 0 were excluded. Of the remaining 513 patients with DFU, 241 (47.0%) were in the D−/I− group, 110 (21.4%) were in the D−/I+ group, 35 (6.8%) were in the D+/I− group and 127 (24.8%) were in the D+/I+ group. Baseline characteristics according to ulcer depth and infection category are presented in Table 1. Peripheral neuropathy was diagnosed in over 90% of patients in all four groups, with no significant difference between the groups (*p*=0.660). There was a significantly higher proportion of diagnosed PAD in the deep DFU groups (D−/I− 38.2%; D−/I+ 36.4%; D+/I− 51.4%; D+/I+ 63.0%; *p*<0.001) and a significantly lower proportion of SGLT2 inhibitor use (D−/I− 29.9%; D−/I+ 29.1%; D+/I− 17.1%; D+/I+ 18.1%; *p*=0.047). The levels of C-reactive protein (CRP) were significantly higher in the infected DFU groups (*p*<0.001). Significant differences between the four groups (Table 1) were also observed for type 1 diabetes (*p*=0.048), ulcer area ≥1 cm^2^ (*p*<0.001), midfoot or hindfoot location of the ulcer (*p*=0.011), ulcer status at follow-up (*p*<0.001) and active ulcer at follow-up (*p*=0.040).

**Table 1:** Baseline characteristics according to ulcer depth and infection categories

Characteristic	D−/I− group (*n*=241)	D−/I+ group (*n*=110)	D+/I− group (*n*=35)	D+/I+ group (*n*=127)	*p* value
Age (years)	64.8±12.3	63.8±13.1	63.3±12.2	67.5±13.6	0.088
Male	184 (76.3)	84 (76.4)	26 (74.3)	95 (74.8)	0.982
Indigenous Australian	11 (4.6)	4 (3.6)	4 (11.4)	7 (5.5)	0.309
Lowest two quintiles for SEIFA^a^	90 (37.8)	46 (42.2)	16 (45.7)	60 (48.0)	0.289
Type 1 diabetes	21 (8.7)	19 (17.3)	4 (11.4)	9 (7.1)	0.048*
Hypertension	179 (74.3)	74 (67.3)	23 (65.7)	93 (73.2)	0.455
Dyslipidaemia	132 (54.8)	56 (50.9)	18 (51.4)	83 (65.4)	0.107
Ever smoker^b^	97 (69.8)	54 (72.0)	19 (73.1)	58 (74.4)	0.908
Dialysis	16 (6.6)	4 (3.6)	2 (5.7)	7 (5.5)	0.733
HF	27 (11.2)	9 (8.2)	5 (14.3)	17 (13.4)	0.587
Prior PCI/CABG	35 (14.5)	17 (15.5)	6 (17.1)	30 (23.6)	0.162
PAD	92 (38.2)	40 (36.4)	18 (51.4)	80 (63.0)	<0.001***
Stroke/TIA	25 (10.4)	8 (7.3)	4 (11.4)	22 (17.3)	0.091
Peripheral neuropathy	229 (95.0)	101 (91.8)	33 (94.3)	120 (94.5)	0.660
Retinopathy	64 (26.6)	27 (24.5)	12 (34.3)	36 (28.3)	0.702
Nephropathy	66 (27.4)	34 (30.9)	12 (34.3)	35 (27.6)	0.782
COPD	19 (7.9)	0	0	11 (8.7)	<0.001***
Depression	36 (14.9)	15 (13.6)	2 (5.7)	15 (11.8)	0.463
Osteoporosis	8 (3.3)	6 (5.5)	1 (2.9)	7 (5.5)	0.672
SGLT2 inhibitor	72 (29.9)	32 (29.1)	6 (17.1)	23 (18.1)	0.047*
GLP1 agonist	60 (24.9)	24 (21.8)	9 (25.7)	28 (22.0)	0.879
Statin	158 (65.6)	73 (66.4)	27 (77.1)	89 (70.1)	0.504
ACEI/ARB	148 (61.4)	68 (61.8)	21 (60.0)	72 (56.7)	0.821
Antiplatelet	101 (41.9)	46 (41.8)	19 (54.3)	66 (52.0)	0.166
Anticoagulant	40 (16.6)	21 (19.1)	4 (11.4)	32 (25.2)	0.145
HbA_1c_ (mmol/mol)	69.5±23.5	73.9±23.7	73.2±28.8	74.2±26.3	0.245
HbA_1c_ (%)	8.5±2.1	8.9±2.2	8.8±2.1	8.9±2.4	
LDL-cholesterol (mmol/l)	1.9±0.8	2.0±1.0	1.8±0.9	2.1±1.1	0.602
eGFR <60 ml/min per 1.73 m^2 c^	82 (35.3)	38 (36.2)	14 (40.0)	47 (37.6)	0.943
CRP (mg/l)	7.2 (3.4–21)	54 (14–145)	22 (5.8–33)	74 (18–152)	<0.001***
Ulcer area ≥1 cm^2^	85 (35.3)	49 (44.5)	26 (74.3)	71 (55.9)	<0.001***
Midfoot or hindfoot	48 (19.9)	23 (20.9)	4 (11.4)	10 (7.9)	0.011*
Ulcer status at follow-up^d^					<0.001***
Not healed	62 (25.9)	30 (27.5)	16 (45.7)	40 (31.7)	
Healed	166 (69.5)	66 (60.6)	15 (42.9)	57 (45.2)	
Amputation	11 (4.6)	13 (11.9)	4 (11.3)	29 (23.0)	
Active ulcer at follow-up^d,e^	84 (35.1)	51 (46.8)	19 (54.3)	57 (45.2)	0.040*

Data are means ± SD, *n* (%) or median (IQR)

^a^SEIFA was categorised into quintiles (where 1 is the most disadvantaged and 5 is the least disadvantaged), according to residential postcode. SEIFA quintile data were not available for six patients (1.2%)

^b^Smoking history data were not available for 195 patients (38.0%)

^c^eGFR data were not available for 16 patients (3.1%)

^d^At time of event or end of follow-up. Information on ulcer status at follow-up was not available for four patients (0.8%)

^e^Includes ulcers that have not healed, concurrent ulcers, recurrent ulcers or new ulcers

*p* values indicate statistically significant differences at *p<0.05, **p<0.01, ***p<0.001

ACEI, angiotensin-converting enzyme inhibitor; ARB, angiotensin II receptor blocker; CABG, coronary artery bypass graft; COPD, chronic obstructive pulmonary disease; PCI, percutaneous coronary intervention; SEIFA, Australian Socio-Economic Indexes for Areas; TIA, transient ischaemic attack

Incident MACE or all-cause mortality occurred in 75 patients (14.6%) and incident MACE alone occurred in 46 patients (9.0%) after a median follow-up of 381 days (IQR 220–551) and 404 days (IQR 228–576), respectively. MACE included 21 (45.7%) hospitalisations for myocardial infarction (of which three were fatal), 20 (43.5%) hospitalisations for HF, and five (10.9%) hospitalisations for stroke (of which one was fatal) or transient ischaemic attack. Baseline characteristics according to the occurrence of MACE and mortality outcomes are presented in ESM Table 2. Comorbidities significantly associated with the occurrence of incident MACE included a prior diagnosis of HF (*p*<0.001), prior coronary artery revascularisation (*p*=0.004), diagnosed PAD (*p*<0.001), prior stroke or transient ischaemic attack (*p*=0.023) and diagnosed nephropathy (*p*<0.001). CRP levels were significantly higher in patients who experienced incident MACE or all-cause mortality (*p*<0.001) and MACE alone (*p*<0.001). Compared with forefoot location of the ulcer, a midfoot or hindfoot location was associated with a significantly higher risk of incident MACE or all-cause mortality (*p*=0.027) and MACE alone (*p*=0.025). Patients with active ulcers at follow-up had significantly higher risk of incident MACE or all-cause mortality (*p*<0.001) and MACE alone (*p*=0.002).

**Fig. 1:**
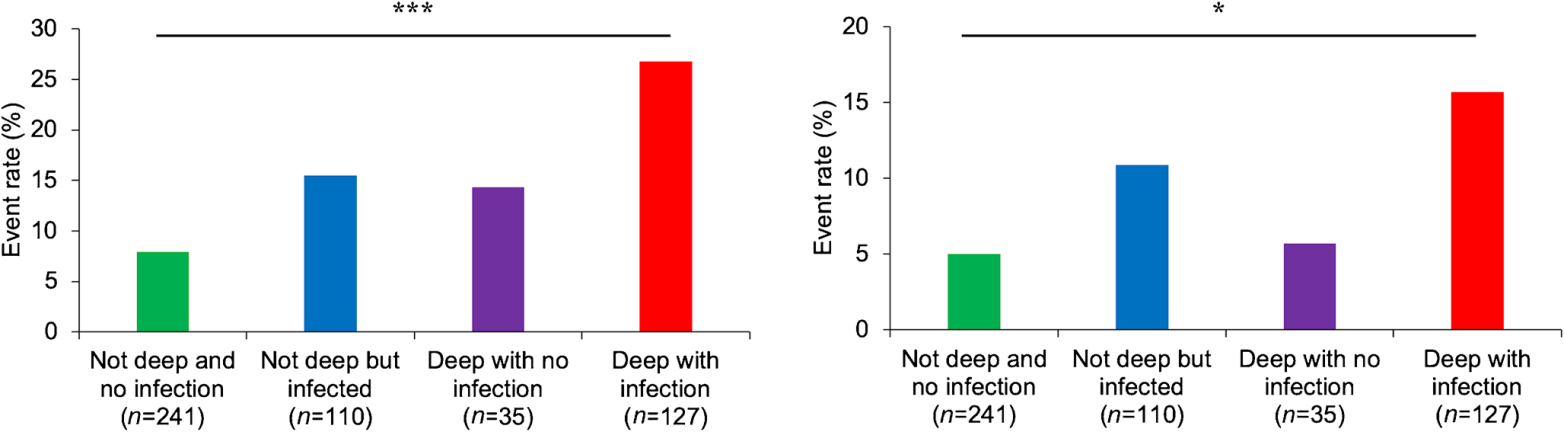
MACE and mortality outcomes according to ulcer depth and infection categories after a median follow-up time of 381 days (IQR 220–551) for MACE or all-cause mortality (**a**) and 404 days (IQR 228–576) for MACE alone (**b**). The *p* values shown were calculated using the logrank test: *p<0.05, ***p<0.001

**Fig. 2:**
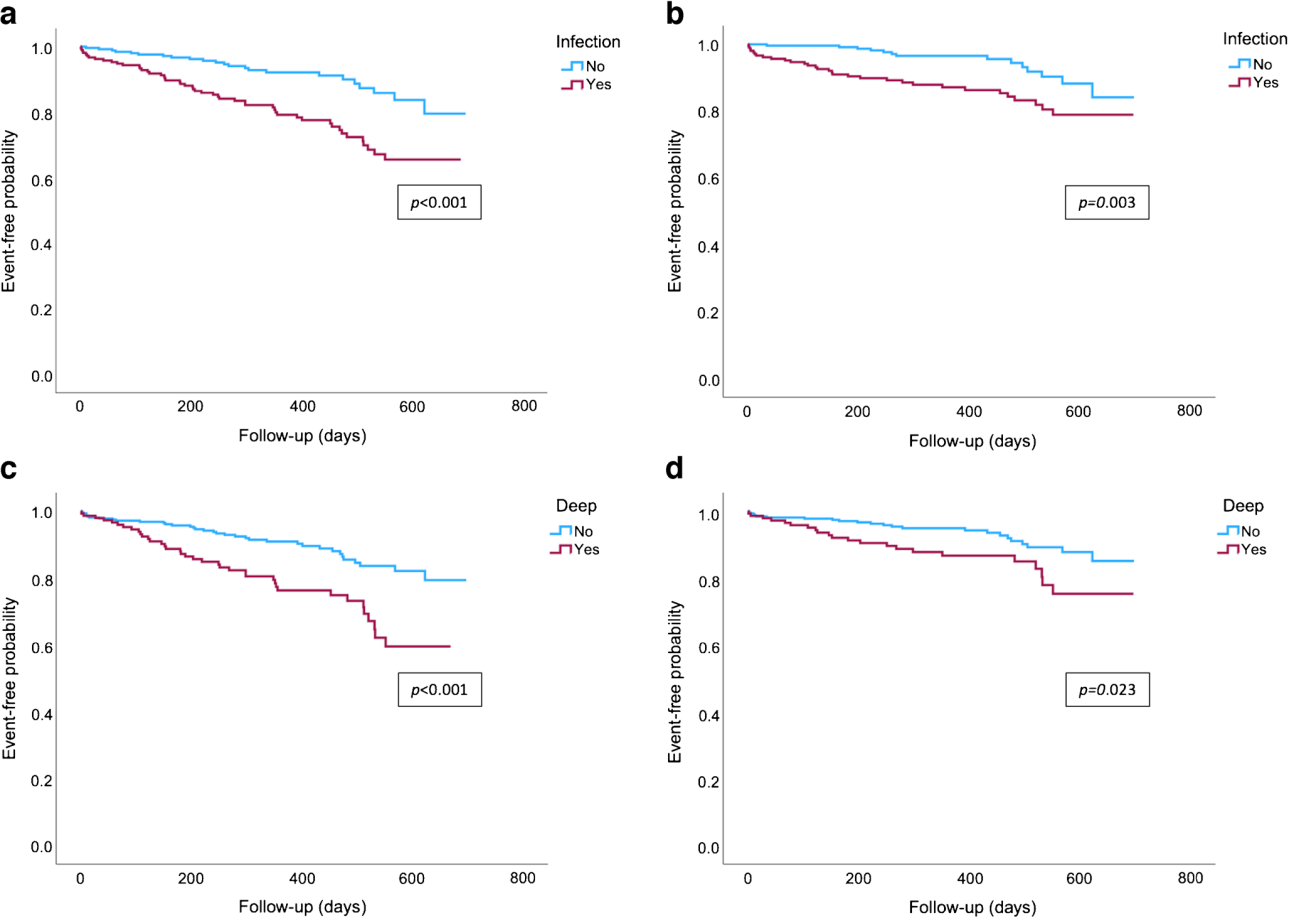
MACE and mortality survival curves according to whether the ulcer is infected (**a**, **b**) or deep (**c**, **d**) for MACE or all-cause mortality (**a**, **c**) or MACE alone (**b**, **d**). The median follow-up time was 381 days (IQR 220–551) for MACE or all-cause mortality and 404 days (IQR 228–576) for MACE alone. The *p* values shown were calculated using the logrank test

The group with deep and infected DFU had a significantly greater proportion of patients who experienced incident MACE or all-cause mortality (D−/I− 7.9%; D−/I+ 15.5%; D+/I− 14.3%; D+/I+ 26.8%; logrank *p*<0.001) or MACE alone (D−/I− 5.0%; D−/I+ 10.9%; D+/I− 5.7%; D+/I+ 15.7%; logrank *p*=0.017), as shown in Fig. 1. Compared with patients with no DFU infection (*n*=276; 53.8%), a greater proportion of patients with DFU infection (*n*=237; 46.2%) experienced incident MACE or all-cause mortality (21.5% vs 8.7%; logrank *p*<0.001) or MACE alone (13.5% vs 5.1%; logrank *p*=0.003) (Fig. 2). Similarly, compared with patients without deep DFU (*n*=351; 68.4%), a greater proportion of patients with deep DFU (*n*=162; 31.6%) experienced incident MACE or all-cause mortality (21.9% vs 10.3%; logrank *p*<0.001) or MACE alone (13.6% vs 6.8%; logrank *p*=0.023) (Fig. 2).

In the Cox regression analyses (Table 2), patients with deep and infected DFU had a significantly higher rate of MACE or all-cause mortality in the unadjusted model (*p*<0.001) and the adjusted models (model 2, *p*=0.001; model 3, *p*=0.004; model 4, *p*=0.006). Similarly, patients with deep and infected DFU had a significantly higher rate of MACE alone in the unadjusted model (*p*=0.024) and the adjusted models (model 2, *p*=0.013; model 3, *p*=0.018; model 4, *p*=0.013). In the final model (model 4), deep and infected DFU was associated with three times the risk of MACE or all-cause mortality (HR 3.03; 95% CI 1.62, 5.67) and MACE alone (HR 3.61; 95% CI 1.59, 8.17) compared with DFUs that were not deep and not infected. Similar findings were observed in Cox regression analyses of deep ulcers or infection as separate covariates (Table 3), but deep ulcer was not significantly associated with MACE alone in model 3 (*p*=0.056).

**Table 2:** Multivariate Cox regression models for ulcer depth and infection categories as a predictor of MACE and mortality outcomes

		Model 1^a^		Model 2^b^		Model 3^c^		Model 4^d^	
Outcome	Group	HR (95% CI)	*p* value	HR (95% CI)	*p* value	HR (95% CI)	*p* value	HR (95% CI)	*p* value
MACE or all-cause mortality	Not deep and no infection	Reference	<0.001***	Reference	0.001**	Reference	0.004**	Reference	0.006**
	Not deep but infected	1.84 (0.95,3.54)		2.13 (1.10, 4.15)		2.09 (1.04, 4.18)		2.00 (0.99, 4.03)	
	Deep with no infection	1.81 (0.68, 4.86)		2.06 (0.75, 5.69)		1.53 (0.53, 4.40)		1.57 (0.53, 4.60)	
	Deep with infection	3.39 (1.94, 5.95)		3.30 (1.84, 5.92)		3.03 (1.66, 5.52)		3.03 (1.62, 5.67)	
MACE alone	Not deep and no infection	Reference	0.024*	Reference	0.013*	Reference	0.018*	Reference	0.013*
	Not deep but infected	2.03 (0.91, 4.52)		2.44 (1.08, 5.51)		2.94 (1.25, 6.93)		2.94 (1.24, 6.99)	
	Deep with no infection	1.13 (0.25, 5.07)		1.53 (0.33, 7.03)		1.40 (0.29, 6.75)		1.65 (0.33, 8.23)	
	Deep with infection	2.97 (1.45, 6.07)		3.38 (1.60, 7.12)		3.21 (1.47, 7.03)		3.61 (1.59, 8.17)	

^a^Unadjusted

^b^Adjusted for age, type 1 diabetes, social deprivation score, hypertension, dyslipidaemia, ever smoking, SGLT2 inhibitors and GLP1 agonists

^c^Adjusted for covariates in model 2 plus atherosclerotic CVD (either CHD, PAD or cerebrovascular disease), HF, nephropathy and a number of other comorbidities (chronic obstructive pulmonary disease, depression and osteoporosis)

^d^Adjusted for covariates in model 2 and 3 plus wound area (≥1 cm^2^) and location (midfoot or hindfoot)

*p* values indicate statistically significant differences at *p<0.05, **p<0.01, ***p<0.001

**Table 3:** Multivariate Cox regression models for ulcer depth or infection as separate predictors of MACE and mortality outcomes

		Model 1^a^		Model 2^b^		Model 3^c^		Model 4^d^	
Outcome	Group	HR (95% CI)	*p* value	HR (95% CI)	*p* value	HR (95% CI)	*p* value	HR (95% CI)	*p* value
MACE or all-cause mortality	Not infected	Reference	<0.001***	Reference	<0.001***	Reference	<0.001***	Reference	0.001**
	Infected	2.40 (1.48, 3.91)		2.46 (1.49, 4.02)		2.40 (1.52, 3.98)		2.31 (1.39, 3.86)	
	Not deep	Reference	<0.001***	Reference	<0.001***	Reference	0.005**	Reference	0.007**
	Deep	2.40 (1.52, 3.77)		2.26 (1.41, 3.64)		2.00 (1.23, 3.24)		2.04 (1.21, 3.41)	
MACE alone	Not infected	Reference	0.005**	Reference	0.002**	Reference	0.002**	Reference	0.001**
	Infected	2.49 (1.32, 4.67)		2.78 (1.46, 5.29)		2.94 (1.51, 5.75)		3.05 (1.54, 6.02)	
	Not deep	Reference	0.026*	Reference	0.016*	Reference	0.056	Reference	0.032*
	Deep	1.94 (1.08, 3.45)		2.11 (1.15, 3.88)		1.85 (0.98, 3.46)		2.09 (1.07, 4.10)	

^a^Unadjusted

^b^Adjusted for age, type 1 diabetes, social deprivation score, hypertension, dyslipidaemia, ever smoking, SGLT2 inhibitors and GLP1 agonists

^c^Adjusted for covariates in model 2 plus atherosclerotic CVD (either CHD, PAD or cerebrovascular disease), HF, nephropathy and a number of other comorbidities (chronic obstructive pulmonary disease, depression and osteoporosis)

^d^Adjusted for covariates in model 2 and 3 plus wound area (≥1 cm^2^) and location (midfoot or hindfoot)

*p* values indicate statistically significant differences at *p<0.05, **p<0.01, ***p<0.001

## Discussion

To the best of our knowledge, this is the first study demonstrating that deep ulcers (reaching muscle, tendon or deeper structures) and/or infected ulcers are associated with an increased risk of MACE, as well as MACE or mortality, in patients with DFU. This study also highlights a relatively high rate of MACE on the background of a high prevalence of major cardiovascular risk factors (or ‘risk enhancers’), including hypertension, dyslipidaemia, microvascular disease and established atherosclerotic CVD, including PAD and prior coronary artery revascularisation. The high rate of MACE is plausible, as the pathogenesis of DFU is multifactorial and is closely associated with underlying microvascular disease (e.g. peripheral neuropathy) and macrovascular disease (e.g. PAD) [31]. Despite this, multivariate analyses adjusting for several baseline comorbidities that are known to portend worse cardiovascular outcomes revealed that deep and/or infected DFU remains an independent predictor of future MACE. These findings provide a potential mechanistic explanation for the observed excess risk of CVD in people with DFU.

DFU represents a significant and highly morbid complication of diabetes, leading to lower-extremity amputation, disability, hospitalisation and substantial healthcare costs globally [32]. Among people with diabetes, the lifetime incidence of DFU ranges from 19% to 34% [33]. Notably, a meta-analysis has shown that five-year mortality approaches 50% in people with DFU, with approximately half of the explainable deaths being attributed to CVD [1]. In addition, a systematic review and meta-analysis demonstrated that people with DFU have a more than twofold higher risk of cardiovascular death compared with people with diabetes without DFU [4]. A higher prevalence of stroke in people with DFU has also been reported [34]. Even in the case of neuropathic DFU, as opposed to ischaemic DFU, CHD is the leading cause of death [2, 3]. Numerous observational studies have demonstrated that DFU is associated with all-cause mortality, an association that persists after adjusting for confounders such as cardiovascular risk factors, pre-existing CVD and nephropathy [2, 35–37]. Despite these findings, few studies have investigated the mechanisms linking DFU to worse cardiovascular outcomes. Concomitant infections, such as osteomyelitis or polymicrobial growth in deep tissue, gangrene, the severity of the DFU and hindfoot location, have been previously shown to be associated with a significantly higher risk of mortality, but their impact on incident MACE has not been reported [10–14].

### Inflammation and infection as a mediator of MACE

We hypothesise that chronic low-grade inflammation, compounded by superimposed infections, further exacerbates the systemic inflammatory response that contributes to an accelerated development and progression of CVD and HF in people with DFU. The complex interplay between inflammatory, immune and metabolic abnormalities associated with DFU and the potential implications on the risk of CVD have been highlighted previously [38]. DFU induces marked upregulation of inflammatory markers such as TNF-α, IL-6 and CRP, which correlate with ulcer severity [23–25]. It is well-established that inflammation promotes endothelial dysfunction, oxidative stress, atherothrombosis and atherosclerotic plaque destabilisation, leading to myocardial infarction or stroke [15]. Moreover, persistent inflammation can lead to myocardial fibrosis and cardiac remodelling, ultimately leading to HF [16]. Clinical trials of anti-inflammatory therapies support inflammation as being a driving force behind CVD; therapies such as canakinumab (a human monoclonal antibody against IL-1β) and colchicine have been shown to significantly reduce MACE in very-high-risk patients [39, 40]. Thus, it is conceivable that inflammation in the context of acute and chronic DFU plays a role in increasing cardiovascular risk over and above the inflammation that is observed in the context of diabetes.

Chronic infections in extravascular locations, such as DFU, may provide a persistent inflammatory stimulus that contributes to the overall inflammatory burden [15]. A somewhat analogous example is periodontitis, which has been shown to increase the risk of CVD [41]. Deep infection of DFU may also introduce a significant bacterial load, often polymicrobial, into the systemic circulation, leading to immune system activation and subsequent inflammation [15]. In addition, there is evidence that infecting microorganisms can directly stimulate vascular inflammation [15]. It has also been shown that acute infections, such as pneumonia, may trigger myocardial infarction, potentially due to the cytokine response causing atherosclerotic plaque rupture and/or as a result of myocardial ischaemia due to supply/demand mismatch during sepsis [42]. In the present study, it is noteworthy that CRP levels were higher in patients diagnosed with infected DFU and those who experienced MACE. Furthermore, heightened inflammation may persist even after resolution of acute infections; this has been highlighted in studies of pneumonia and may also be relevant to DFU [43]. These observations underscore the need for further research to elucidate infection-related factors in the context of DFU that potentially contribute to the heightened cardiovascular risk.

### Clinical implications

The management of people with DFU is complex, necessitating attention to multiple inter-related, multi-system issues. Despite the recognised high cardiovascular risk among this population, there remains a significant gap in their cardiovascular care [44]. Our findings underscore the critical need to assess cardiovascular risk and intensively manage risk factors in patients with DFU, especially if deep infection is present. Promisingly, an observational study demonstrated that implementing an intensive cardiovascular risk factor screening programme and utilising risk-reducing therapies, such as statins, in people with DFU was associated with a reduction in five-year mortality from 48% to 27% [45]. However, there is still a need for a deeper understanding of the underlying mechanisms linking DFU and CVD. ‘Metabolic memory’ for example, can occur due to chronic hyperglycaemia, leading to epigenetic changes that may affect DFU healing, inflammation, infection risk and vascular function [46]. Given the findings of our study, it may be further postulated that anti-inflammatory approaches have a role in reducing cardiovascular risk in people with DFU. To modify chronic inflammation in the context of diabetes, strategies optimising smoking cessation, dietary modification, physical activity, weight loss and the use of statins, GLP1 agonists and SGLT2 inhibitors, may be required [26]. Whether people with DFU derive greater cardiovascular benefits from anti-inflammatory therapies, such as colchicine, requires investigation. Furthermore, ulcer duration has been associated with an increased risk of mortality [47]. Strategies that promote the resolution of inflammation, such as earlier treatment and/or therapies to accelerate wound healing, could potentially reduce cardiovascular risk but would require further studies. Timely and improved access to multidisciplinary DFU care may reduce infections and major amputations, and improve healing [31].

### Limitations and strengths

Limitations of this study include its observational nature and relatively modest sample size. The sample size may have resulted in this study being underpowered to detect significant differences between groups (e.g. D−/I− vs D+/I−). Sex-disaggregated analyses were not performed due to the relatively small sample size for each ulcer depth and infection category. The results of multivariate analyses should be interpreted with caution due to the low absolute number of events. Outcome data could only be accessed from public hospital records; thus MACE and mortality rates may have been underestimated. Furthermore, the cause of death could not be ascertained for many patients, which may also have led to an underestimated rate of MACE. In addition, no specific biomarkers were available to separate inflammation from infection. We also did not have information on other factors that may impact cardiovascular risk such as lipoprotein(a) levels, physical activity levels, family history, recreational drug use, cognition and measures of vascular function [46]. As a single-centre study in a major metropolitan area, our findings may not be generalisable to other contexts with differences in care delivery. For example, SGLT2 inhibitors have prognostic benefit in people with diabetes but were less often prescribed in those with deep DFU [48]. This may reflect concerns relating to increased lower-extremity amputations based on the CANVAS trial and guideline recommendations [49, 50]. However, the current findings add to the small body of evidence examining infection of DFU and cardiovascular outcomes. Strengths of this study include the analysis of standardised data regarding wound characteristics, the presence or absence of clinical infection, and the presence or absence of comorbidities that were prospectively collected in consecutive patients for an electronic registry. Moreover, data were collected from the year 2022 onwards, reflecting contemporary practice.

### Conclusion

In conclusion, deep and/or infected DFU is associated with a significantly increased risk of future cardiovascular events. This supports the concept that inflammation and infection can drive adverse atherosclerotic CVD and HF outcomes, expanding the evidence base to include patients with DFU. Given our findings, comprehensive cardiovascular risk assessment and intensive risk factor modification should be prioritised in patients with DFU, particularly when deep infections are present. Our novel findings also suggest the existence of potential mechanistic pathways that should be evaluated in future mechanistic research and larger studies.

## Supplementary Information

Below is the link to the electronic supplementary material.

ESM Tables (PDF 125 KB)

## Data Availability

All relevant data are contained within the manuscript.

## Abbreviations

CRP: C-reactive protein
D−/I−: DFU not deep and no infection
D−/I+: DFU not deep but infected
D+/I−: DFU deep with no infection
D+/I+: DFU deep with infection
DFU: Diabetes-related foot ulceration/ulcer
HF: Heart failure
GLP1: Glucagon-like peptide-1
MACE: Major adverse cardiovascular events
PAD: Peripheral arterial disease
SGLT2: Sodium-glucose cotransporter 2
SINBAD: Site, ischaemia, neuropathy, bacterial infection, area and depth
