# Supplementary material for: Increased risk of major adverse cardiovascular events in patients with deep and infected diabetes-related foot ulcers
Source: Diabetologia. 2024 Nov 7;68(2):460–70. doi: 10.1007/s00125-024-06316-z (PMC11732954; doi:10.1007/s00125-024-06316-z)
Supplement: Supplementary file 1 — ESM Tables (PDF 125 KB) [file 125_2024_6316_MOESM1_ESM.pdf]

ESM Table 1 – SINBAD System

**Category**	**Definition**	**Score**
Site	Forefoot<br>Midfoot and hindfoot	**0**<br>**1**
Ischaemia	Pedal blood flow intact: at least one palpable pulse<br>Clinical evidence of reduced pedal flow	**0**<br>**1**
Neuropathy	Protective sensation intact<br>Protective sensation lost	**0**<br>**1**
Bacterial infection	None<br>Present	**0**<br>**1**
Area	Ulcer <1 cm^2<br>Ulcer ≥ 1 cm^2	**0**<br>**1**
Depth	Ulcer confined to skin and subcutaneous tissue<br>Ulcer reaching muscle, tendon or deeper	**0**<br>**1**
Total possible score		**6**

ESM Table 2 – Baseline characteristics according to occurrence of MACE and mortality outcomes

**Characteristic**	**No MACE or mortality (*n*=438)**	**MACE or mortality (*n*=75)**	***p* value**	**No MACE (*n*=467)**	**MACE (*n*=46)**	***p* value**
Age (years)	64.3±12.5	70.2±13.8	<0.001***	64.8±12.7	68.5±13.9	0.091
Male	328 (74.9)	61 (81.3)	0.228	353 (75.6)	36 (78.3)	0.686
Indigenous Australian	22 (5.0)	4 (5.3)	0.782	22 (4.7)	4 (8.7)	0.278
SEIFA lowest two quintiles[a,b]	178 (41.2)	34 (45.3)	0.503	191 (41.4)	21 (45.7)	0.580
Type 1 diabetes	48 (11.0)	5 (6.7)	0.259	49 (10.5)	4 (8.7)	1.000
Hypertension	308 (70.3)	61 (81.3)	0.050*	331 (70.9)	38 (82.6)	0.091
Dyslipidaemia	237 (54.1)	52 (69.3)	0.014*	259 (55.5)	30 (65.2)	0.203
Ever smoker[b]	192 (70.8)	36 (76.6)	0.419	209 (71.3)	19 (76.0)	0.619
Dialysis	21 (4.8)	8 (10.7)	0.042*	26 (5.6)	3 (6.5)	0.737
HF	37 (8.4)	21 (28.0)	<0.001***	43 (9.2)	15 (32.6)	<0.001***
Prior PCI/CABG	65 (14.8)	23 (30.7)	<0.001***	73 (15.6)	15 (32.6)	0.004**
PAD	173 (39.5)	57 (76.0)	<0.001***	197 (42.2)	33 (71.7)	<0.001***
Stroke/TIA	43 (9.8)	16 (21.3)	0.004**	49 (10.5)	10 (21.7)	0.023*
Peripheral neuropathy	413 (94.3)	70 (93.3)	0.744	440 (94.2)	43 (93.5)	0.744
Retinopathy	108 (24.7)	31 (41.3)	0.003**	123 (26.3)	16 (34.8)	0.219
Nephropathy	106 (24.2)	41 (54.7)	<0.001***	123 (26.3)	24 (52.2)	<0.001***
COPD	22 (5.0)	8 (10.7)	0.054	25 (5.4)	5 (10.9)	0.128
Depression	60 (13.7)	8 (10.7)	0.474	62 (13.3)	6 (13.0)	0.965
Osteoporosis	16 (3.7)	6 (8.0)	0.086	17 (3.6)	5 (10.9)	0.021*
SGLT2 inhibitor	118 (26.9)	15 (20.0)	0.205	121 (25.9)	12 (26.1)	0.979
GLP1 agonist	112 (25.6)	9 (12.0)	0.011*	114 (24.4)	7 (15.2)	0.161
Statin	291 (66.4)	56 (74.7)	0.159	312 (66.8)	35 (76.1)	0.199
ACEI/ARB	272 (62.1)	37 (49.3%)	0.037*	271 (58.0)	25 (54.3)	0.630
Antiplatelet	190 (43.4)	42 (56.0)	0.042*	206 (44.1)	26 (56.5)	0.107

Anticoagulant	77 (17.6)	20 (26.7)	0.063	85 (18.2)	12 (26.1)	0.193
HbA_{1c} (mmol/mol)	72.4±24.2	69.0±24.3	0.275	72.3±24.4	67.9±23.0	0.243
HbA_{1c} (%)	8.8±2.2	8.5±2.2		8.8±2.2	8.4±2.1	
LDL-cholesterol (mmol/L)	2.0±0.9	1.9±1.1	0.528	2.0±0.9	1.8±1.1	0.426
eGFR <60 ml/min per 1.73 m^2 [b]	130 (30.8)	51 (68.0)	<0.001***	151 (33.5)	30 (65.2)	<0.001***
CRP (mg/L)	16 (4.9-53)	91 (16.2-163)	<0.001***	19 (5.1-56)	100 (11-180)	<0.001***
Ulcer area ≥1cm^2	193 (44.1)	38 (50.7)	0.288	209 (44.8)	22 (47.8)	0.689
Midfoot or hindfoot	66 (15.1)	19 (25.3)	0.027*	72 (15.4)	13 (28.3)	0.025*
Ulcer status at follow-up[b,c]			<0.001***			0.062
Not healed	114 (26.3)	34 (45.3)		129 (27.9)	19 (41.3)	
Healed	278 (64.1)	26 (34.7)		284 (61.3)	20 (43.5)	
Amputation	42 (9.7)	15 (20.0)		50 (10.8)	7 (15.2)	
Active ulcer at follow-up[b,c,d]	163 (37.6%)	48 (64.0%)	<0.001***	182 (39.3%)	29 (63.0%)	0.002**

Data are means ± SD, *n* (%) or median (IQR)

[a]SEIFA was categorised into quintiles (where 1 is the most disadvantaged and 5 is the least disadvantaged), according to residential postcode

[b]Smoking history data not available for 195 (38.0%) patients, SEIFA quintile data were not available for 6 patients (1.2%), eGFR data were not available for 16 patients (3.1%) and ulcer status or active ulcer at follow-up was not available for 4 patients (0.8%)

[c]At time of event or end of follow-up.

[d]Includes ulcers that have not healed, concurrent ulcers, recurrent ulcers or new ulcers.

p values indicate statistically significant differences at *p<0.05, **p<0.01, ***p<0.001

ACEI, angiotensin-converting enzyme inhibitor; ARB, angiotensin II receptor blocker; CABG, coronary artery bypass graft; COPD, chronic obstructive pulmonary disease; CRP, C-reactive protein; eGFR, estimated glomerular filtration rate; GLP1, glucagon-like peptide-1; HF, heart failure; LDL, low-density lipoprotein; PAD, peripheral arterial disease; PCI, percutaneous coronary intervention; SEIFA, Australian Socio-Economic Indexes for Areas; SGLT2, sodium-glucose cotransporter 2; TIA, transient ischaemic attack.
